# Observations on the Use and Functions of the Salivary, Lachrymal, and Other Glands in the Human System

**Published:** 1841

**Authors:** H. H. Hayden

**Affiliations:** Dental Surgeon, Baltimore.


[ From the Medical Repository of 1818, Vol. 4th, New Series. ]

### OBSERVATIONS

### *On the Use and Functions of the Salivary, Lachrymal, and other Glands in the Human System—by* H. H. Hayden, Esq., *Dental Surgeon, Baltimore.*

Having taken a comparative view of different nations and tribes of mankind, and noticed in a brief manner, the prevalence of good or bad teeth among them, and the probable effect of habits and actions in producing, or promoting decay in the teeth, when considered relatively, as to climate; having, in a more particular manner, pointed out the effects, and its operations in promoting the premature decay of teeth so generally in the United States, viz: by the general prevalence of glandular affections; I shall, in the next place endeavor, not only to prove my position as it respects the glands, but to point out and identify what (at least in my opinion) constitutes the universal cause of decay of human teeth.

In treating of the subject of Infantile Dentition, I have assigned as the cause of most of the evils attendant on that period, or at least during the prevalence of the symptoms, that of morbid or acrid secretions: I have there pointed out the existence of this agent, and its operation on the infant alveolar processes and teeth; also, its prevalence and operations, as an effect of certain diseases, particularly the meazles, small pox, and high inflammatory or malignant fevers, and in such a manner, as not to leave a doubt of its influence or capacity to act upon and destroy bone.

Therefore, I feel no hesitation in saying, that the decay of human teeth is occasioned almost universally by an acrid, or morbid secretion from the mouth and gums.

Before I proceed to describe the manner in which this agent is employed in the destruction of the human teeth, and of its operations in the endless variety of cases in which it occurs, it seems necessary, to establish the fact beyond a doubt, that there is such a thing as morbid or acrid secretions in the mouth; otherwise my views, however plausible, cannot be sustained. In order to which, it becomes necessary likewise, to take a cursory physiological view of the human mouth, and some of the neighboring parts.

The mouth, forming a cavity, very large and capacious in itself, is lined with a membrane or skin of the most delicate texture or fineness throughout the entire cavity, covering every inequality of its internal surface from the pharynx to the edge of the lips. The gums, which invest the edge of the superior, as well as inferior jaw, and which surround all the teeth likewise in both jaws, are very irritable and easily inflamed as well from an internal as an external cause—are covered with the same delicate skin or membrane.

The neighboring parts which I have to notice, are the different glands situated in and about the mouth, and which will be duly attended to in course.

The mouth, therefore, (since it is admitted that into all cavities in the human system* there is a constant secretion) must be considered as subject to healthy or morbid secretions at all times, and that in an eminent degree.

In order that this opinion may not rest entirely upon my assertion for its support, I shall endeavor to procure testimony of the most unquestionable kind; and since it is unnecessary to multiply authorities or quotations, I shall confine myself principally to the opinions of Mr. John Bell, and John Hunter.

In Mr. Bell's System of Anatomy, (Vol. 4, page, 22, written I believe by Charles Bell) he observes that, " We have to recollect that every part of the body secretes, and that every surface is a secreting surface; and that even the surface which is produced by an incision no sooner ceases to bleed, than a secretion begins;" (and I will venture to add, that the progress of healing in the incision, will depend almost entirely on the healthy or morbid state of the fluid secreted, and not on the admissions of, or exposure to, external air."—(See Lubbue & Allen.) This is a circumstance, extensive in itself as it relates to the system, and which, if examined attentively, will afford much interesting and useful speculation.) But to return to the subject again; Mr. Bell, in speaking of the lacteals and lymphatics of the intestinal canal, (vol. 4, page 327,) observes, " We have remarked also, that while *every surface of the body secretes*, it is at the same time an absorbing surface; and finally, that while we chiefly contemplate the intestinal canal as imbibing and receiving the nourishment, we must not forget that it is also a *secreting surface* of the first importance to the economy."

Mr. Bell, in treating of absorption, (in vol. 4, page 299) says, " This interstitial membrane is elastic, and being cellular, to allow of motion, its surface is *bedewed* with *serous exudation*." " This fluid is perpetually passing from the extremities or sides of the lymphatic arteries or capillaries, into the cellular membrane, and upon *all the cavities* of the *body*." Further, " The pores, or vessels from which this fluid *exudes*, are called exhalents; and their action is, no doubt, as completely secretion as that which produces the fluid, which in our wisdom, we call more perfect secretions.†"

In describing the inner coat of the œsophagus, Mr. Bell says, (vol. 4,

* See Shaller, Phisy. p. 18, article 43.

† See also Bell's Anat. vol. 4, p. 28.

page 42,) "It in every respect resembles the *lining membrane of the mouth.* This inner coat has an exhaling surface, like the rest of the body, with *particular glands* to *secrete and pour out the mucous* which lubricates the passage for the food."

Whether Mr. Bell's opinions of the mode or manner in which secretion is performed be most correct, viz: by the exhalents, or that of Dr. Hunter, who supports the opinion "that the fluids of cavities were collected by transudations, and not thrown out by exhalents," is not for me, in the present instance, to determine. It will suffice that I adduce sufficient and satisfactory evidence of the existence of fluid secretions in the mouth, and other cavities, of which, exclusive of Mr. Bell and Hunter's opinions, there is abundant testimony. It remains yet to prove, that fluid secretions are at any time, or under any circumstances, unhealthy, so as to constitute an acrid, or otherwise morbid secretion. Of this, independent of what daily experience teaches us, I think we have sufficient proof in the opinion or writings of the reputable author before mentioned: who says (vol. 4, page 300,) that, "By disease, the fluids in the cavities and cellular membrane is *altered.* In dropsy, for example, the fluid of the abdomen loses the property of coagulating on mere exposure; it comes to resemble more the serum of the blood: this were sufficient proof that the collection is not owing merely to the diminished absorption, but, that there is a *change* of *action* in the *vessels of the peritoneum, pleura, pericardium,* &c." Again, "An inflammation of the vessels will throw out a fluid more coagulable, and which, in a high degree of action, will form a *film* of *coagulable lymph,* or *even pus,* on the surface. But in a state the reverse of inflammation—such for example as the debility following inflammation, a serous effusion will be poured out having little tendency to coagulate."

Having noticed the opinions of Messrs. Bell and Hunter, which I think is alone sufficient to authorize my proceeding to an explanation of the effects of acrid secretion, or its operation in the destruction of the human teeth; yet, as there are some important collateral correspondencies which it is necessary to examine, in order to concentrate the amount of evidence on the point at issue, I must beg the favor of an indulgence in another small, but not unimportant digression.

"If," as Mr. Bell says, "an inflammatory action of the vessels will throw out a fluid more coagulable, and which, in a high degree of action will form a film, or coagulable lymph, or even *pus* on the *surface*" (of the cellular membranes and cavities)—if this acrid or morbid secretion is capable of corroding the coats of the membranes, or lining of cavities; of producing *ulceration,* and *causing* a variety of *aggravated evils* yet to be enumerated; has Nature, in the plenitude of her wisdom, made no provisions against an attack so serious and so subversive of the beauty, order and utility of her noblest works? It will doubtless be answered, that the absorbents are a provision against it. To this I shall not, at present reply; but will first examine the salivary glands of the mouth, particularly as was observed in the preceding page.

In attempting a superficial view of the probable intentions and use of some of the glands in the human system, I shall necessarily be led a little out of the bounds of my province ; yet it is not my intention to treat of them as a subject generally; or could I entertain a wish to do it, I have not the vanity to think myself, in the smallest degree, capable of exploring the secret labyrinths of a system which has at different periods engaged the combined talents of the medical world, and which is acknowledged to be at the present time involved in doubt and mystery.*

Well might Mr. Bell style the glandular systems, and doctrines of absorption, the most beautiful and interesting in the whole economy—for the more the mind is engaged in contemplating this mysterious plan, as well as that of almost all of nature's works ; the more it sees of the wonderfully existing harmonies, and surprising adaptations, manifested throughout the whole ; the more it is inclined to believe that there is not an organized atom in animated nature, that does not beam with intelligence at every pore.

It is asserted by most physiological writers on this subject, that the salivary glands are *necessary* to digestion—that this is the *intention*, *use*, and *operation* of the fluid secretions from them.

A very natural and reasonable conclusion, I admit, as much so as that the water in a marsh attenuates the mud ; or that the water of a brook emptying into a river, increases the quantity.

But I beg leave to ask if they really were intended for this purpose ; are they really necessary in that operation ? If they were *intended* for that purpose, why do they discharge their contents in a direct contrary direction from the orifice of the pharinx : as the maxillary, sublingual, and in fact, the parotid glands ? The situation of the salivary ducts, from whence the saliva is discharged into the mouth, seem to indicate that they were not intended, or at least exclusively, to assist in digestion ; for, had they discharged their contents more immediately into the œsophagus, it would have, perhaps, obviated that excessive waste of this fluid which prevails in many instances, without any visible effect. If intended to soften down the food in mastication, and to facilitate digestion, might we not as reasonably expect to see the maxillary and sublingual ducts discharging their contents in the neighborhood of the parotid ducts, and like them falling more immediately on the food in mastication ; or between the dentes molares and cheek of the inferior jaw, to be acted on by either or both, the masseter and buccinator muscles ?

In reply to this, it may be said that if they were situated on the out side of the jaws, they would be much more liable to obliteration from cuts or wounds, as is sometimes the case with the parotid ducts. This will be admitted ; but it would be not only rare, but truly unfortunate, if they should be obliterated on both sides.

A much more probable reason presents itself in opposition to such an arrangement. It would not have answered so well the views and intentions of the great Author of our frail system, as the present distribution of those important organs.

Secondly, are the salivary glands essential to the digestion of our food ? for, if for that purpose, it follows that they are necessarily so, therefore

* See Bell and Hallen, page 25, vol. 4, page 10, 298—'9.

cannot be dispensed with without producing some material derangement in the economy of the system; for it will seldom be found that nature, in the execution of her plans, employs anything unnecessarily, although at first it may appear so.

I shall in the first place take some notice of the probable quantity of fluid that is secreted from the salivary glands, together with the quantity discharged from the *pancreas.*

It is said by *Helvetius* that a soldier of the guards received a cut of a sabre across the cheek which laid open the parotid duct: the wound healing on the inside of the cheek, occasioned a constant discharge outwardly from the parotid duct. At the time of his repasts there flowed from the orifice an abundance of saliva; insomuch that during dinner, which is short in the Hotel Dieu, it moistened several napkins.

Sabatier mentions that a soldier moistened three napkins during a short repast, by the saliva which escaped from one of the salivary ducts of Stenorr, which remained open from the effects of a wound.

Gavard mentions having seen at the Hotel Dieu, a man affected with a similar complaint, and who lost at a meal in the space of ten minutes, at least two ounces of saliva.

It is also said that the salivary glands have been known to discharge twelve ounces in an hour. See Haller's Phy. p. 293.

It is likewise said that half an ounce an hour is constantly discharged and swallowed.*

Therefore, it appears agreeable to the case mentioned by Gavard, that if two ounces of saliva are discharged in ten minutes (and which is confirmed by Haller) the quantity discharged in twenty-four hours would amount to two hundred and eighty-eight ounces.

But, it will be considered, that their quantity was discharged at the time of a repast, and therefore at a time when the glands were subject to the greatest action of the muscles which promote the discharge of the saliva—consequently not a proper time by which to ascertain the probable aggregate quantity discharged in twenty-four hours.

If we admit the twelfth part of the above quantity to be secreted (including the hours of repast, at which time much the greatest quantity is discharged) and extend it to sixteen hours only, for very little is discharged from them during sleep, the quantity will amount to sixteen ounces.

If we next consider the pancreas, which is three times as large as all the salivary glands of the mouth,† and admit a proportionable quantity of fluid secreted, it will amount to forty-eight ounces in sixteen hours, and probably much more, if we were to form a conclusion on the comparative size of the Ductus Virsungi, and the salivary ducts.

This quantity added to the salivary discharges, amounts to sixty-four ounces in sixteen hours; exclusively of the glandular secretions of the mouth, œsophagus and stomach. A quantity that far exceeds, at least in most instances, the amount of food received in twenty-four hours, without taking into view the quantity of drinks of different kinds that are received into the stomach during that time.

* See Haller's Phy. p. 318. † Ibid. p. 335.

If we next consider the nature of the food which constitutes our daily subsistence, as to its being fluid or solid, we should be able to form a more correct opinion of the probable quantity of juices required to digest it. If the salivary juices are *necessary* to promote digestion, how is it that those who are in the constant habit of chewing tobacco, opium, &c. from the time they arise in the morning, until they go to rest at night, (hours of repast excepted) and who scarce swallow a drop of the saliva; yet are strong, robust, and uncommonly healthy, and in general more exempt from disease than those on the contrary who do not indulge those habits, (at least those who chew tobacco.) There are exceptions to this rule in some instances I admit, where, as is supposed, it has probably induced a dyspepsy, and even phthisis.

But I am inclined to believe, that much of the result in many of those instances may be ascribed, with truth, to the effect that it has on the mind. For in many cases of this kind that occur, they are persons who have recently commenced the habit of chewing tobacco ; or if not so, they are not only in the habit of spitting constantly, but under perpetual apprehensions of swallowing a drop for fear of creating nausea. This produces a dryness in the fauces, and an almost constant but involuntary effort to swallow without allaying, in any degree, the increasing propensity. At length it becomes a source of vexation ; a constant but almost insensible teazing of the mind, until, without reflecting on the cause, a wasting of the substance is manifest, although, at the same time the due secretions and evacuations of the system are regularly performed.

I think I may venture to assert, that a sharp-pointed feather permanently fixed in a person's clothes in such a manner as to goad him constantly in some tender, or irritable part, would produce not only a wasting of flesh, but a real dispepsy, and perhaps something worse.

Diseases are often produced in this manner merely from the sharp-pointed remains of a decayed tooth, at which the tongue is incessantly at work, and which at length deprives the patient of every comfort ; and fever is induced, and often symptoms not only unfavourable, but extremely obstinate.

It is the constant but slow teazing the mind that will often, from various circumstances, produce surprizing effects in the constitution.

It was thus that the lion was wrought up to a phrenzy, not by the pains occasioned by the goadings of a simple gnat, fixed in his nostril; but by the incessant teazing his *royal intellect.*

If the salivary juices are necessary to promote the digestion of the grosser kinds of food in use among us, can it be considered as necessary for the Arabs, and many of the tribes of Africans, * as well as many of the Turks, Egyptians, Hindoos, &c., who live almost constantly on boiled rice, dates, and milk ? If so necessary in performing those important functions, how do not only those who chew tobacco, opium, &c., dispense with the saliva ; but those who from falls, blows, cuts, &c., have unfortunately had their under teeth knocked out, and lip so lacerated that the saliva is constantly running from their mouth, without the power of re-

* See Park's Travels.

straining it ; and also those who are sometimes long subject to fistulous holes under the chin that extend into the mouth ; and through which the saliva is incessantly discharging except during sleep?

Lastly, there is a period in infancy, at least for several weeks, when there is no visible discharge of saliva in the mouth of the infant, for there is little or none running from the mouth ; and they are seldom seen or known to swallow, except when at the breast, or fed. Nor do they for many months, except at the times abovementioned, acquire the faculty of swallowing with facility ; not even until they have several teeth ; during which time they begin to partake, more or less, of grosser food than milk ; it is then we see the saliva constantly running from their mouths, without ever making an effort to swallow it, or even restrain it except laid on their backs, at which time it collects on the pallate and obliges them to swallow. If it is so necessary, how can they for the first eighteen or twenty months dispense with so much; and yet thrive so fast and enjoy such a measure of health? I admit, that it is wisely and *necessarily* provided by the bounteous gift of nature ; that it is indispensible ; that without it we should become loathesome to ourselves and all those around us ; yet I must be indulged in the belief that it was intended also for other purposes equally important, and such as are explained hereafter.

The human mouth being considered as subject at all times, under various circumstances, not only to healthy but to morbid secretions, which, under certain degrees of action are liable to form a sizey, acrid, and sometimes incrassated mass, or collection of fluid in this cavity ; and which is often rendered more so by the free access of atmospheric air. It seems evident that in this state it is unfit to be received into the stomach, or be re-absorbed.

Hence we see this wise provision, this copious flow of saliva into the mouth, doubtless with the intention to correct the acrimony, by diluting the unhealthy secretions, and thereby preventing its injurious effects in the mouth, rendering it easy and safe if carried into the stomach, and easy of being absorbed.

That they were intended, at least in part, for this purpose admits not a doubt in my mind ; that they are necessary for that purpose, must appear evident from an almost infinite variety of circumstances.

In a state of perfect health, and during the hours of sleep, when there is scarcely any discharge of the salivary juices ; the natural secretions of the mouth are sufficient to lubricate it, and render it free from any degree of fœtor, or inconvenience when we awake.

But on the contrary, when any derangement of the economy exists from an impurity of the blood, or a vitiated state of the fluids, we are sensible of the most unpleasant effects in the mouth when we awake from sleep.

But as soon as the action of the muscles on the salivary glands promotes the discharge of their juices, this inconvenience, even without eating or drinking, is in a short time removed, and the mouth is rendered comfortable.

If a fever prevails, during which the salivary discharges are almost entirely suspended, this inconvenience, in proportion to the acuteness of the fever, will be increased also, until the patient's situation is rendered extremely uncomfortable from the filthy accumulation of matter in the mouth, that is often so acrid as to produce a soreness throughout the whole cavity, and even the skin on the tongue cracks open in different places and bleeds.

If attenton is paid to the period of infantile dentition, we see the importance of the salivary juices displayed (in this view) in a striking manner.

If the irritation is increased so far as to excite a moderate ptyalism, we see the salvia flowing in abundance from the mouth of the little sufferer; but if fever ensues, the salivary secretions are at once suspended almost, and the tongue becomes furred over, and often, before it terminates, a general appearance of aptha is manifested in the mouth. (I would not be understood in the present instance as meaning that the aptha and sometimes ulceration is produced in consequence of morbid secretions.) As soon as the fever subsides, and the salivary glands resume their functions, the incrassated secretions, and unnatural appearance of the tongue is removed, and a more healthy action is re-established in the mouth.

Many authors in writing on the different diseases, incident to mankind, and their symptoms, have noticed also, the appearance of the tongue. But I know of no one that has marked its appearance, in disease and health, more particularly than Mr. Abernethy in his "observations on the disorders of health;" in which are mentioned, a number of cases that would afford, I think, much real support to the opinions I have advanced on the use of the salivary glands, could I feel myself fully at liberty to comment upon, or question the opinions of so reputable an author.

Such, however, is the effect of some of the diseases to which we are subject, in promoting acrid secretions in the mouth, and other parts of the system, that the necessity of such a provision as the salivary glands must be acknowledged as indispensably necessary, or, however well we might support ourselves under the operations of the disease, the effects would probably be such as to render us odious in the sight of each other, while we lived to linger through a miserable existence.

Such for instance, would be the effects of a severe attack of the meazles.

I may be thought perhaps, to have given much too great a latitude in the opinion, particularly as it relates to the disease above mentioned.

But such are the effects of this disease in the mouth, which I have often witnessed, that, were it not for the copious discharge of saliva, I should not be surprized to see the mouth eaten into holes, as well as the stomach, and the whole of the intestinal canal. Therefore, since in this point of view, there is such a striking analogy between the eye and the mouth, particularly as to the effects of the above disease, I shall make a few remarks upon it.

It is generally supposed that the secretions from the glandula lachrymalis were intended to wash away chips, sticks, or moates that accidentally fall in upon the eye; to lubricate it, and also to supply us with tears when we are disposed to weep or cry.

If this were true, we might expect to see millers, ash-men, chimney sweepers, and others who work in the dust, with their eyes always full, or running with tears, which is not the case. But as soon as the acrid effects of smoke are felt upon the eye, we see the saliva (for it differs none in quality) or tears poured forth, to prevent its inflaming, or otherwise injuring the coats of the eye. Thus it is on removing the skin from an onion. In proportion to the quantity or strength of the acrid effluvia received from the onion into the eye, so likewise will be the discharge of the tears, until the fluid is sometimes seen almost to stream from the eye. Such also is the effect of vegetable alkali, insomuch that every reflecting mind will admit, that were it not for this beautiful provision of nature, and which is necessarily so, inflammation would ensue, and in all probability terminate in total blindness.

Notwithstanding the numerous glands which nature has supplied for the purpose of lubricating the eye, the mouth, the œsophagus, and in fact, almost every part of the system; yet they are alike, secreting surfaces; therefore, subject during the prevalence of diseased action in the system to the effects of acrid or morbid secretions as well as healthy.

Thus we see, in common inflammation of the eye, during the night, or the hours of sleep, when, like the salivary glands, there is little or no discharge from the lachrymal gland, the eye-lids in the morning are so completely agglutinated that it is not without difficulty that it can be opened; and when it is, the coats of the eye are so susceptible that even the light is painful to them. But as soon as the due secretions from the glands take place, the irritation is in some degree allayed, and the pain relieved.

It would be easy to point out in numerous instances, the serious evils and unhappy consequences that would inevitably result to the eye, as well as mouth, from a total deficiency of this important fluid if it were necessary. But the effect of some of the diseases to which we are subject, on the eyes, as well as mouth, affords such conclusive testimony in favor of the opinion I have advanced that it would seem needless to pursue it any farther. Such as those of the inflammatory kind, catarrhal affections, small pox, and particularly the meazles, on which it is needless to comment, since it must be admitted, by all who reflect on the nature of a severe attack of that complaint, that were it not for these organs, we should present ourselves an awful spectacle to every beholder.

This opinion will, I think, be more strongly verified in the course of the following observations, which relate, not only to the important use of the lachrymal juices, correcting the acrid secretions of the eye; but also to the like important use of the salivary juices in often correcting the highly septic state of the mouth, from morbid secretions.

It is not my intention, in the present instance, to enter into a pathological disquisition of the disease called the scurvy, as it relates either to the mouth, or system generally. Suffice it to say, that the cases of scurvy in the mouth mostly complained of, are almost entirely local, and proceed from an accumulation of tartar about the teeth, irritating the gums, and rendering them often much diseased; and from causes producing acrid secretions which lodge about the teeth, irritating the investing membranes, &c.

The result of these are diseased, flabby gums, surcharged with black, impure blood, swollen and bleeding at the slightest touch—discharging at times a thin, acrid fluid, which, with the other secretions of the mouth, are carried into the stomach, corroding and irritating, (in some instances) the entire passage of the alimentary canal.

The number of persons is composed of a very large proportion of society, at least in this country, and it is said by a late writer* that at least three fifths of the inhabitants of large Cities in France, are subject to this complaint.

I shall, in the course of this subject, take some farther notice of the frequency of this affection of the mouth, and of the cause of its prevalence.

But since there is a striking similarity in this and the more inveterate scurvy prevailing at sea, and on long voyages, as it respects acrid secretions in the mouth, I shall take more particular notice of the latter in order to prove, or at least point out the use and necessity and importance of the salivary juices in correcting acrid secretions.

Whatever may constitute the cause of this dreadful disease, it is enough for the present purpose to know that some of its symptoms are a melancholy dejection of spirit ; a lassitude ; a stiffness in the joints, and great difficulty in breathing ; the gums soon after begin to swell, itch, and bleed, having an unusual, livid redness—all of which symptoms indicate, together with yellowish complexion, and gradually growing darker ; a highly morbid or depraved state of the fluids. An universal dryness of the skin is a certain indication of a feverish habit (which prevails excepting the last stage of the disease) during which the functions of the salivary glands are almost totally suspended ; hence the distressing dryness of the mouth and throat.

In this state the acrid secretions of the mouth are left to commit their ravages, by lodging round or about the teeth under the gums ; the investing membrane of the teeth is then irritated, inflamed, the teeth become loose and drop out ; the gums become ulcerated and phagedenic, while the whole system is verging fast to dissolution.

Can acrid secretion be denied here ? or the want of healthy salivary secretions, or some wholesome substitutes to correct the acrimony and attenuate the incrassated mucous in the mouth ? At least I think not !

In the next place, as a further illustration of the use and intention of the salivary juices in correcting the acrid secretions of the mouth, I would call the attention to that state in which persons die by starvation, or famine.

Here we see displayed the effects of the one, through a deficiency of the other ; and the *tragic finale* ending in *death* the most lingering, and most to be dreaded.

In this unhappy state, when the support of nature is cut off or withheld, and the blood, from a deficiency of nourishment† tending to an alkaline disposition, and at length approaching a putrid acrimony ; ‡ this acrid secretion manifests itself not only in the mouth, but in the thoracic and abdominal viscera by the most violent pains (at least in the former) which terminate sometimes in actual madness.

* See Laforgue on the diseases of the mouth.
† See Haller's Physiology, p. 314, or article 439.
‡ See Bell's Anatomy, vol. 4, p. 64.

In this situation the breath becomes fœtid, and the secretions of the mouth excoriate the gums and cheeks, irritating the investing membrane of the teeth, until they become loose and drop out; while the seasonable aid of the salivary juices is cut off by a dreadful fever, which suspends, as in almost all cases, the operations of the salivary glands, which otherwise would have corrected the acrimony, and lessened in some degree the miseries that are on such an occasion, seated in the mouth.

Lastly, it is said that the inhabitants of Biledulgerid (a province in the north of Africa) are subject, at certain seasons of the year, to an inveterate scurvy in their gums which is attributed to their eating of dates, which is almost the only vegetable they have that is eatable, and which grows in abundance in that country.

I would ask, why are not the inhabitants of the other different provinces in Africa, or of Barbara, afflicted with this complaint, who live mostly on dates; and also the Arabians and the lower order of the Egyptians and Turks? It is also said of those people that they are very subject to inflammations in the eyes, which is attributed to the reflection of the sunbeams from the white hard soil.

I would likewise ask, why are not the inhabitants on the borders of Zaara and the great desert of Arabia, or those who are in the habit of travelling in those regions, afflicted with those complaints? If so in some instances, I should ascribe it to another cause.

Why are not the inhabitants of the cold regions of the North, afflicted with this disease of the eyes, who are subject not to beholding white sand, but a boundless region of snow, which operates more severely by straining the eye than the whitest sand at present known?

Mention is also made by numbers, and particularly by M. Volney in his travels in Egypt and Syria, of the prevalence of diseases of the eyes, and of blindness among the Egyptians, and which is frequent among the Syrians, and which he attributes to the "corrupted humours" which are afforded by the peculiar nourishment which they take, and to the saline quality of the air, which he considers as constituting the "first principles of those disorders." These humours being "diverted from the ordinary channels by habitual perspiration, they fly to the exterior parts, and fix themselves where they find the least resistance." They therefore naturally attack the head, because the "Egyptians, by shaving it once a week, and covering it with a prodigiously hot head-dress, principally attract to that the perspiration."

It appears to me that Mr. Volney need not have gone so far in search of the cause of this complaint; or if he considered onions which he mentions as conducing to this complaint, and of which those nations consume great quantities, he would have been careful from notions of humanity, to caution his countrymen to abstain from the use of garlic, which is equally calculated to produce opthalmies.

It is a well known fact that all persons who perspire very freely, complain of a soreness and smarting under the eyes, which is in general attributed to the heat of the sun, or weather, and "the sweat running into the eyes." It is also a well known fact, that in a profuse or constant perspiration the salivary secretions are much diminished, and sometimes, almost or quite suspended, the mouth and throat parched up for want of something to moisten it. It may be observed too, that when the salivary secretions are diminished, the lachrymal excretions are likewise ; that, although the thirst of the mouth and throat may be allayed by drink, the eye cannot drink except indirectly.

Hence, the constant secretion of the coats of the eye, receiving some change or modification by the access of external air, are left to irritate the coats of the eye and thereby produce that unpleasant burning sensation experienced by those who perspire freely, merely from a deficiency of the lachrymal secretion to correct its operation.

This seems to be strengthened by the relief experienced by those who in this situation wash their eyes in cold or fresh water.

It is observed by the same author, that this disease prevails more on the sea coast than elsewhere ; without doubt, the water which they drink or use, possesses a saline quality.

To these circumstances may be attributed (I think) most of the inconveniencies which they suffer from this complaint.

In the first place, the saline quality of the water naturally promotes thirst, of course a diminution of salivary secretions ; the same is experienced in the air, although we may not be so sensible of it.

In the next place, this monstrous head dress, promoting and keeping up a constant perspiration in the head, tends to diminish the salivary and lachrymal secretions, till at length, the secretions of the eye and surrounding surfaces becoming more and more acrid, inflammation ensues, which terminates in a distressing opthalmia, or total blindness. This opinion receives additional support from further observations of the same author, viz : That while the natives are subject to this disease, strangers are almost exempt—for a very good reason—strangers are not in the habit of wearing the enormous turban.

" It will appear more probable, that the excessive perspiration of the head, is a principal cause, (thereby attracting the humours to the eyes, as in his opinion,) when we reflect that the ancient Egyptians who went bare headed, are not mentioned by physicians as being much afflicted with opthalmies."

But to return to the inhabitants of Bilidulgerid, it will be recollected that this large extent of country is barren and mountainous : That during several months at least, the air is intensely heated ; so much so, that for six months in the year, almost every fountain or source of water is exhausted or dryed up.*

* It is worthy of observation that, although in the different provinces of Barbary the plague rages with dreadful fury, yet the inhabitants of Bilidulgerid are exempt from it, so that it is not even known among them, although a constant intercourse is kept up with the neighboring provinces. Thus an abundance of water has its bad effects as well as a deficiency, which no doubt, contributes largely in producing this disease, by promoting an habitual perspiration, particularly about the head.

Therefore, it is from the want of water that they are reduced to this unhappy situation, and not from eating dates, or looking at white sand.

It is, doubtless, from a continued privation of this refreshing liquid to quench their parched throats, that the resources of nature in the system are likewise exhausted ; and as in famine, the acrid secretions begin their operations on the eyes as well as mouth ; producing inflammation in the one, and corroding the internal surface of the other, irritating the membranes of the teeth ; they at length become loose and all drop out. This seems but the commencement of the dreadful scene ; for in proportion as the dissolution of the fluids progresses, the secretions become more acrid, and being swallowed and carried into the stomach, without the diluting aid of the saliva to correct, or lessen its acrimony, thereby disordering its functions and increasing the evils by producing general disease, which terminates in the most distressing symptoms of certain dissolution of both fluids and solids ; commencing at the mouth and eyes and spreading over the body, rendering the patient loathsome to himself and all who are around him.

Here, if I am correct, we see at once displayed in a striking manner, the use and importance of the salivary juices in diluting and correcting the acrid secretions of the mouth, and also of the eyes.

Here, too, we see presented (when viewed in this light) a fabric whose internal structure discloses a system in the glandular and absorbent economy, comprizing such symmetry, such endless correspondencies and beauty, as beggars the sublime genius, and boasted talents of man to imitate, in all his various modifications, and shows it worthy of only Him from whose hands it came, and whose works are not only inimitable, but inscrutable.

Having pointed out what I consider one of the most important uses of the salivary and lachrymal glands, and their secretions ; having noticed the correspondence between the absorbents and those glands, I beg leave at least to skim over the surface of this exhaustless fountain, which affords such pleasing speculation, in order to trace the correspondencies into the general system ; for they are not confined to the mouth and eyes.

If the fluid secretions in different parts of the human body are susceptible of being changed into coagulum, or even pus, on the surface of the different cavities and membranes ; if at any time they are morbid, become acrid, capable of irritating and inflaming the parts, so as to render the salivary or any other juices necessary to correct the acrimony, or dilute or dissolve the coagulum so as to render it *easy* of *absorption*, in any one instance ; it certainly must be necessary throughout the whole system.

That this is a rational conclusion, I think will be made to appear from a variety of corresponding circumstances, not only interesting in themselves, but worthy of the most deliberate investigation.

That the thoracic and abdominal viscera are subject to morbid excitement, particularly the latter, from acrid secretions, I am inclined to believe, from a variety of circumstances, not, however, grounded on *my own experience.*

Mr. Bell says, (vol. 4. p. 29)—"I have seen in the high state of inflammation of the membrane (the peritoneum) pus formed upon the surface without ulceration, and therefore, probably from the same exhaling surface; and coagulable lymph lying in flakes upon it. (Note.) The increase of the serous discharge forms the common ascites; but whenever the *natural secretion*, or *exudation* from the peritoneum *is altered*, *adhesions* are *apt* to *form*."

Does inflammation produce this morbid secretion and adhesion? or does an acrid secretion (or natural secretion *altered*) produce an inflammation in the coats of the peritoneum, the result of which is an adhesion? Does the fluid injected, in the operation for hydrocle, produce an inflammation and consequent adhesion or union of the tunica vaginalis, and tunica albuginica? or is there a pre-existing inflammation which only requires the healing qualities of a little wine to heal it over, thereby producing a union of those membranes? I confess I feel a corresponding conviction relative to both; but shall suspend my opinion.

If there is an increase of the halitus or aqua pericardica in disease,*—if it "concretes into villi, and cellular substance"—or is, at times, "somewhat viscid,"† and "immensely increased," is it probable that the secretions at any time become acrid, and capable of exciting inflammation and adhesion of the pericardium and the heart? To this I do not feel competent to answer. But, admitting those secretions in the abdominal cavity; are there no provisions made to correct their quality, or counteract their effects in a certain degree, and under particular circumstances? Mr. Haller makes mention of "a peculiar kind of glands, called conglobate,"‡ which are of an oval form, or shape following the course of the blood-vessels throughout almost every part of the system—of which he observes—"Of what service these glands are to the lymphatics, is not yet well known."

Are those the glands that perform the office of pouring out a thin fluid like the salivary juices, for the purpose of diluting and attenuating the viscid secretions in the different cavities of the system, rendering them easy of absorption; and also, for correcting its acrimony? That these are organs alloted to this purpose, must appear evident on mature reflection: that they are destined of *necessity*, must appear equally obvious: that they are even admitted, though in an indirect manner, by different authors, is equally as certain.

Mr. Haller's writings seem to carry this idea in several instances, though he has not declared it in any one that I am apprized of; as (in his Physy. p. 25, article 56,) for instance; after observing that the use of those glands were not known, he remarks that, "It is, however, rendered probable by late experiments, that in these glands *some kind* of *fluid* is *prepared* which is *mixed* with the *lymph*.

* See Bell, Anatomy, vol. 2, p. 52 and 54.

† See Haller's Phy. p. 35.

‡ See Haller's Phy. p. 24 and 25.

His opinion of the use of the blood of the spleen, in diluting the sluggish blood of the liver, (whether correct or not*) seems to imply that the fluids, at times, require the operation of some agent to facilitate their circulation, and prevent obstructions.

Mr. Bell likewise gives us much reason to conclude, that such a provision is made (if I comprehend him) in the system; and that this is their use we are left to infer from his own words, in vol. 4th, p. 57, when speaking of the third cellular coat of the stomach, he says, "It is in this coat that anatomists have found small dlundular bodies lodged, *especially* towards the extremities or orifices of the stomach."

Again, at page 58, when describing the internal coat of the stomach, which he says "has not been so fully examined, and is not so *perfectly understood* as the analogous appearance of the intestines," he observes that, "*Glands* are also described as opening upon the inner surface of the stomach; and those who have not been able to see these glands, which are seated in the third cellular coat, yet believe in their existence from analogy; while others observe foramina toward the *upper* and *lower* orifice of the stomach, which they *suppose* to be the openings of ducts or glands." "These, however, which I believe to be merely folicles, are themselves the secreting surface, and not the ducts of the proper round glandular-bodies; *at the same time* it must be admitted, that in disease, as if magnifying and giving size to the structure of the stomach, they shew a *glandular* and *tuberculated structure*." (This is certainly not "a poverty of imagination," but "a genius at twistical reasoning scarce to be met with.")

From the following, I think we may reasonably infer what is the use of those glands, since their existence seems to be admitted.

"It is almost superfluous to observe, that the gastric juice has no power of acting on the coats of the stomach during life; whether this be owing to the property in the living fibre, or resistance to the action of the fluid, or *that there* is a *secretion bedewing* the *surface* which *prevents* the action—it is not easy to say."† If for a moment we admit that those organs at the two orifices of the stomach, operate as salivary glands, pouring out a thin aqueous fluid, to correct or attenuate the secretions of the stomach; can it be owing to a suspension of their functions or discharges, that the natural secretions of the stomach become acrid, and causing those distressing pains experienced in a state of starvation, or famine, and a variety of other cases?

From all of the above circumstances, and many more that might have been adduced, I think it must be uniformly admitted, that there is, not only a necessary, but really an established system of (salivary) glands throughout the whole system, and for the necessary and express purpose of diluting viscid secretions, and correcting their acrimony; thereby preventing inflammation in all the different cavities and cells in the human body.

* Haller's Phy. p. 339.
† See Bell's Anat. vol. 4, p. 61.

This opinion not only receives additional support; but is at the same time, beautifully illustrated in a circumstance that follows.

I have observed that it is during the action of the muscles particularly, that the salivary discharges are promoted; and that during a state of rest, there is but little or no discharge from those glands, either in the mouth or eyes—hence such an unpleasant taste in the mouth in the morning; hence also the eyes are often glued up during the night.

It is frequently by contemplating the works and operations of nature, that the most important and latent truths are discovered and brought to light, which would otherwise have remained in obscurity. It is also certain that in many instances analogy is a much surer guide to truth, than the most refined systems in nature.

Hence I am inclined to resort to the following circumstance as a strong collateral testimony, in support of the opinion or subject under consideration.

Whoever is disposed to remark, will find that there is not a human being, or a four-footed animal, (and also many other species of living creatures) existing in a state of health, but what is in the habit of extending its limbs and body, or stretching as it is called, after having remained any length of time at rest, and particularly so after indulging any length of time in sleep. This is common, and may be observed in every animal from the sucking mouse to the sagacious elephant, and also in every human being. It is often observed that this is an indication of thriving; and that it is also necessary in order to stretch the muscles that have become *relaxed*, by a suspension of action.

The one I admit is correct; but I should consider it more properly an indication of health; for where there is a healthy excitement, there is always, I believe, an increased secretion; consequently stimulating to this peculiar action in the man or animal.

But is it probable that it is necessary to stretch the muscular fibre that is already relaxed, from a state of inaction? Or can it be probable, that it is necessary to throw the muscular fibre into a state of tension, to qualify it for an immediate state of inaction again, by lying down at his ease immediately, as is the case with man? Or for animals to stand for hours in one position without moving?

Is it not singular that to a man, or animal that has laboured through a sultry day, in which a copious perspiration has been constantly kept up, his muscles and limbs fatigued and relaxed, should feel no disposition to stretch himself, or extend his limbs in the morning when awake? On the contrary, feels himself as much fatigued almost as when he lay down, though in perfect health? If it is necessary to stretch the limbs, or throw the muscles into a state of tension in order to re-establish their tone, after a few hours of relaxation, is it not equally singular, that little or no disposition to stretch is manifested in a sick animal that has remained in that state for days?—Or a man who has laboured under a violent fever, for the same length of time?

If it is to answer that purpose only, is it not singular, indeed, that man who enjoys an uninterrupted repose through the night, and indulges himself in stretching in the morning when he awakes, should feel an increasing but involuntary propensity to stretch, the more he lounges in idleness through the day, which is invariably the case when in health ?

Rather let it be considered as an inherent principle, endowed by nature, as not only necessary but indispensible to health, both in men and animals, and to preserve us from disease.

It is in a state of rest that the functions of all those that are denominated salivary glands are suspended ; while at the same time the exhalents are performing the act of secretion by the action, or power of the vital force. Hence the necessity, when we awake from sleep, of this general spasmodic or muscular action, by which the glandular system is compressed, and the contents discharged to dilute and correct the secretions ; thereby preventing morbid excitement which might otherwise ensue by being too long retained in a viscid or otherwise acrimonious state.

